# Characterization of the complete chloroplast genome of *Chenopodium quinoa* Willd

**DOI:** 10.1080/23802359.2017.1403866

**Published:** 2017-11-15

**Authors:** Kangyu Wang, Li Li, Shaokun Li, Honghua Sun, Mingzhu Zhao, Meiping Zhang, Yi Wang

**Affiliations:** College of Life Science, Jilin Agricultural University, Changchun, China

**Keywords:** *Chenopodium quinoa*, chloroplast genome, HomBlocks, phylogenomics, phylogenetic relationship

## Abstract

*Chenopodium quinoa* Willd is the main traditional food of Inca aboriginal, which was a native grain in South American andes mountains. It has more than five thousand years of edible and cultivation history. Quinoa is rich of comprehensive nutritional value, therefore, be called as the mother of grain. In this study, the complete chloroplast genome of *Chenopodium quinoa* Willd was determined by the sequencing of PCR fragments. The complete chloroplast genome of *C. quinoa* Willd was 151,169 bp in length and displays a typical quadripartite structure of the large (LSC, 83,576 bp) and small (SSC, 18,107 bp) single-copy regions, separated by a pair of inverted repeat regions (IRs, 24,743 bp each). It harbours 120 gene species, including 87 protein-coding genes, 29 transfer RNA and 4 ribosomal RNA gene species. The overall nucleotide composition was: 31.2% A, 31.5% T, 19.0% C, and 18.3% G, with a total G + C content of 37.3%. Phylogenetic relationship analysis shows that *C. quinoa* closely related to *Chenopodium album*.

*Chenopodium quinoa* Willd. (Chenopodiaceae), commonly known as quinoa, belongs to the family Amaranthaceae which serves as the staple food of the Andean communities. The large variety and relative high content of nutrition make it an important crop with the potential to contribute to food security worldwide which is confirmed by Food and Agriculture Organization of the United Nations (FAO) (Bazile et al. 2015). The adaptability and resistance in many abiotic stresses allow it to become a cultivated crop worldwide (Yasui et al. 2016). Although it has a long history of cultivation, the biological research still at an early stage. The genome of quinoa had just released (Jarvis et al. 2017), which bring the research of quinoa into a new world. In this study, we used the method of whole genomic sequencing to get the chloroplast genome of quinoa, in order to provide information for the study of the origin of crops in evolution.

The specimen of *C. quinoa* was isolated from Jilin Agricultural University quinoa test field in Changchun, Jilin, China (125.40E; 43.82N) and the DNA of *C. quinoa* was stored in Jilin Agricultural University College of Life Science (No. JLAUCLS1). The *C. quinoa* complete chloroplast genome was preliminarily annotated using the DOGMA (Dual Organellar GenoMe Annotator) online program (Wyman et al. 2004), with default settings to identify protein-coding genes, rRNAs and tRNAs based on the Plant Plastid Code and BLAST homology searches. The secondary structures of transfer RNA (tRNA) genes were identified by using the program tRNAscan-SE (Lowe and Eddy 1997) or through manually visual inspection. The graphic map of the complete mitochondrial genome was drawn by using the online software OrganellarGenomeDRAW (Lohse et al. 2007). The annotated chloroplast genome was submitted to GenBank database under accession No. MF805727.

The chloroplast genome sequence of *C. quinoa* is a closed-circular molecule of 151,169 bp in length, which is almost the same with the *Chenopodium album* chloroplast (152,167 bp). It displayed 37 typical gene sets which observed in this plant chloroplast, including 17 PCGs species, 18 tRNA genes (one for each amino acid, two each for Valine, Asparagine, Serine and Threonine, three each for Leucine and Arginine, three each for Methionine), and 2 genes for ribosomal RNA subunits (*rrn*S and *rrn*L). The chloroplast organization of *C. quinoa* is compact, encoding 15,607 bp functional regions (including D-loop region).

We selected other 72 related complete chloroplast genomes from GenBank to assess the phylogenetic relationship between them. The genome-wide alignment of all chloroplast genomes was done by HomBlocks (https://github. com/fenghen360/HomBlocks). The alignment was analyzed by PhyloBayes version 3.2 (Lartillot et al. 2012) under the CAT-GTR + Γ model. Two independent MCMC analyses were run for 10,000 cycles in PhyloBayes. The convergence was checked based on time-series plots of the likelihood scores using Tracer (http://tree.bio.ed.ac.uk/software/tracer/). The first 5000 cycles were discarded as burn-in, and the remaining trees were summarized to obtain Bayesian posterior probabilities (BPP). The final tree was represented and edited using FigTree version 1.4.0. As shown in Figure 1, the phylogenetic positions of these 72 chloroplast genomes were successfully accorded with full BPPs in almost all nodes. As expected, *C. quinoa* showed the closest with *Chenopodium album*.

**Figure 1.**
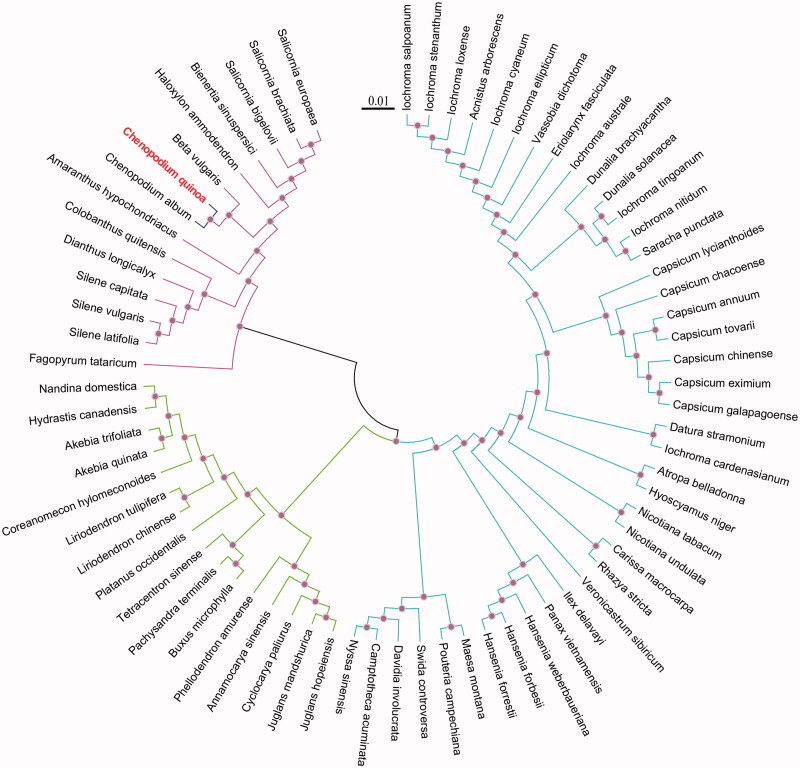
The phylogenetic tree of 72 complete chloroplast genomes which yielded by Bayesian analysis. The scale bar indicates the number of nucleotide substitutions per site.
